# A theory of unusual anisotropic magnetoresistance in bilayer heterostructures

**DOI:** 10.1038/s41598-023-27530-6

**Published:** 2023-01-06

**Authors:** X. R. Wang, C. Wang, X. S. Wang

**Affiliations:** 1 Physics Department, The Hong Kong University of Science and Technology, Clear Water Bay, Kowloon, Hong Kong China; 2 HKUST Shenzhen Research Institute, Shenzhen, 518057 China; 3 William Mong Institute of Nano Science and Technology, The Hong Kong University of Science and Technology, Clear Water Bay, Kowloon, Hong Kong China; 4 Center for Joint Quantum Studies and Department of Physics, School of Science, Tianjin University, Tianjin, 300350 China; 5 School of Physics and Electronics, Hunan University, Changsha, 410082 China

**Keywords:** Materials science, Nanoscience and technology, Physics

## Abstract

The observation of magnetoresistance (MR) varying with the rotation of magnetization in the plane perpendicular to the electric current is an important discovery in spintronics in recent years. The famous conventional anisotropic MR (AMR) says that the resistance of a polycrystalline magnetic material must depend on magnetization component along the current direction only, thus cannot account for this newly observed unusual AMR (UAMR). This UAMR leads to the notion of the spin-Hall MR (SMR) in the famous SMR theory. However, the SMR theory may only explain UAMR observed in heavy-metal/magnetic-insulator bilayers, not other types of bilayers. Here, we present a two-vector theory that can explain not only all existing experiments on the unusual angular dependence of longitudinal and transverse resistivity when the magnetization rotates in three mutually perpendicular planes, but also how three amplitudes of MR angular oscillation are related to each other. The theory is very general and its correctness depends only on the assumption that the magnetization and interfacial field are the only vectors affecting electron transport besides of other scalar variables such as the temperatures and impurities. Experiments that can test this theory against the SMR theory are also proposed.

A dramatic development during the debate of the transverse spin Seebeck effect^1–5^ is the discovery of an unusual anisotropic magnetoresistance (UAMR) in bilayers that consist of either one non-magnetic film and one magnetic film or two magnetic films at nanometer scales. The ubiquitous UAMR occurs in the non-magnetic polycrystalline metallic-film^3–13^, no matter whether it is a heavy metal with strong spin-orbit coupling or not as long as it is highly susceptible to magnetism, and no matter whether the magnetic layer is insulating or not. UAMR exists also in a magnetic polycrystalline metallic-film^14^ in contact with a non-magnetic insulating layer. In comparison with the well-known usual anisotropic magnetoresistance (AMR) that says resistance depends only on the magnetization component along the current, UAMR depends not only on two magnetization components perpendicular to the current, but also on them differently.

In order to appreciate the unusualness of UAMR, let us summarize its main experimental features^3–14^ in a compact form. Denote the interface of a bilayer heterostructure as the *xy*-plane and current along the ${\hat{x}}$-axis, the longitudinal resistivity and transverse resistivity take the forms of $\rho _{xx}=\rho _y+\Delta \rho _1 m_x^2+\Delta \rho _2 m_z^2$ and $\rho _{xy}= Rm_z+\Delta \rho _3m_z^3+\Delta \rho _1 m_xm_y$, where $\vec{m}$ is the unit vector of magnetization $\vec{M}$. $\rho _y$, $\Delta \rho _1$, $\Delta \rho _2$, $\Delta \rho _3$, and *R* are constants whose physical meaning are clear. $\rho _y$ is the longitudinal resistivity of the heterostructure when the magnetization is along the *y*-direction. In addition to the term of $\Delta \rho _1 (\vec{m}\cdot {\hat{j}})^2$ for usual AMR and planar Hall resistance^15^, $\Delta \rho _2$ is the newly discovered UAMR that people cast wrongly as $\Delta \rho _2 [\vec{m}\cdot ({\hat{z}}\times {\hat{j}})]^2=\Delta \rho _2 m_y^2$^5^ although it is mathematically the same as $\Delta \rho _2 m_z^2$ after replacing $m_y^2$ by $1-m_z^2$, where ${\hat{j}}$ denotes the unit vector of the current. *R* and $\Delta \rho _3$ are the amplitudes of one-fold and three-fold angular dependence of unusual planar Hall resistance accompanied the UAMR^3–13^. *R*-term is normally known as the anomalous Hall effect. In terms of above terminology, the usual AMR corresponds to $\Delta \rho _2=\Delta \rho _3=0$.

Spin-Hall magnetoresistance (SMR) theory^16^ provides the popular explanation of UAMR in a bilayer of one non-magnetic heavy metal film and one magnetic insulating film. Due to the spin-Hall effect, a charge current in the heavy metal generates a *y*-polarized spin current propagating toward heterostructure interface. This spin current, in turn, induces a charge current opposite to the original current in the heavy metal through the inverse spin-Hall effect such that the current is reduced and resistivity of the heavy metal increases by a factor of $(1+\theta _{SH})^2$, where $\theta _{SH}$ is spin-Hall angle. The spin current impinged on the magnetic insulator is completely reflected at the heterostructure interface in the ideal scenario when the spin current polarization is collinear with the magnetization of the magnetic insulator such that spin current in the heavy metal vanishes and its resistivity is reduced. Spin current is completely absorbed by the magnetic insulator when its magnetization is perpendicular to the spin current polarization such that above mentioned spin-current-enhanced resistivity prevails.

Although the picture of spin polarized electron transmission and reflection at a heterostructure interface in the SMR theory is opposite to that of tunnelling-magnetoresistance theory, the justification is that polarized electrons exert no torque on a collinear magnetized insulator and cannot pass their spin angular momentum to the insulator such that spin current cannot but reflect back to the heavy metal. SMR theory may be able to explain the UAMR in bilayers of heavy metals with magnetic insulators, but it would be unnatural to explain similar results of bilayers of one heavy metal and one magnetic metal because the assumption of above reflection and transmission of spin current by a magnet is not consistent with physics picture of giant magnetoresistance and tunnelling magnetoresistance. According to giant and tunnelling magnetoresistance, it is easier (harder) for a polarized current to pass through a magnet when its magnetization is parallel (anti-parallel) to the current polarization than that when the magnetization is perpendicular to the incoming electron spins. By the same argument of spin-current enhanced resistivity, SMR theory should predict, for example, $\rho _{xx}$ to be different when the current reverses its direction. Of course, this is not what was observed in experiments. Indeed, challenges of SMR theory for UAMR have already been recognized by many people^5,8,14^ for bilayers of one magnetic metal layer and one either magnetic or non-magnetic insulating layer. In summary, a successful theory that can fully account for observed UAMR in various types of bilayers is yet to be developed. This problem, as well as other subtle ones, motivates this study.

In this paper, we show that UAMR of a bilayer is the natural outcome of polycrystalline metal(s) whose resistance depends on two vectors. One is the magnetization of the bilayer, and the other is an interfacial field perpendicular to the heterostructure interface.

Following the convention in most studies of UAMR, unit axis vectors ${\hat{x}}$ and ${\hat{z}}$ denote respectively the in-plane current direction and the direction perpendicular to the heterostructure interface. The metals of the heterostructure are polycrystalline and heterostructure resistance depends on the magnetization $\vec{M}$ because they are either magnets or magnetized through magnetic proximity effect. All experiments with UAMR show, from thickness dependences of transport properties, that resistance is affected by the heterostructure interface. Theoretically, there exists always charge transfer across the interface because materials on the two sides of the interface have different chemical potentials. This charge redistribution will always create an interfacial potential. The width of the potential well is sensitive to carrier density, and could be order of micrometer in semiconductor and typically nanometer for metals. The potential will inevitably change the electron properties at the interface. In turn, the resistance of a bilayer should be affected by the interfacial potential that provides an effective local electric field perpendicular to the interface. Thus, it is natural to expect that an effective field along $\vec{n}={\hat{z}}$ can affect electron transport and the magnetoresistance of the heterostructure should be a function of both $\vec{M}$ ($\vec{m}$) and $\vec{n}$, as well as other material parameters that are not important for universal forms of UAMR.

For the linear response, the electric field $\vec{E}$ in response to an applied current density $\vec{J}$ in the heterostructure must be

1 $$\vec{E}=\overleftrightarrow {\rho } (\vec{m}, \vec{n}) \vec{J},$$

where $\overleftrightarrow \rho (\vec{m}, \vec{n})$ is the resistivity tensor of rank 2 that depends on microscopic properties of the heterostructure and parameters that defines its thermodynamic state. No matter how complicate the microscopic interactions that a heterostructure might be, the tensor form can only come from the order parameters that characterize the macroscopic states of the system. In the absence of an external magnetic field, $\vec{m}$ and $\vec{n}$ are the only available vectors that can be used to construct tensor $\overleftrightarrow \rho$. Thus, $\overleftrightarrow \rho$ should be the linear combination of $\vec{m}\vec{m}$, $\vec{m}\vec{n}$, and $\vec{n}\vec{n}$^17,18^. Each of the three Cartesian tensors is not irreducible^19^, and can be decomposed into irreducible forms of a scalar, a vector, and a traceless symmetric tensor. Thus, from $\vec{m}$ and $\vec{n}$, it is possible to construct three vectors and three traceless symmetric tensors of ranks 2. They are $\vec{m}$, $\vec{n}$, $\vec{m}\times \vec{n}$, $\vec{m} \vec{m}-1/3$, $\vec{m} \vec{n}+\vec{n}\vec{m}-2\vec{m}\cdot \vec{n}/3$, and $\vec{n}\vec{n}-1/3$, where *M* is the magnitude of magnetization $\vec{M}$. Thus, with these angular dependent terms together with a scaler term, the electric field $\vec{E}$ due to $\vec{J}$, after grouping similar terms and using $\vec{J}$ perpendicular to $\vec{n}$, must take the following generic form

2 $$\begin{aligned} \vec{E}&=\rho \vec{J}+ \vec{J} \times (B_1 \vec{m} + B_2 \vec{n}+ B_3\vec{m}\times \vec{n}) \\ {}&\quad +A_1(\vec{J}\cdot \vec{m})\vec{m} + A_2(\vec{m}\cdot \vec{n})\vec{J}+A_3(\vec{m}\cdot \vec{J})\vec{n}, \end{aligned}$$

where $\rho$ $B_1$, $B_2$, $B_3$, $A_1$, $A_2$, and $A_3$ are parameters that depend on *M* and $\vec{m}\cdot \vec{n}=m_z$, which is the only scalar associated with the direction of $\vec{M}$, as well as other material parameters that have nothing to do with the direction of $\vec{M}$. Thus, the longitudinal resistivity and the transverse resistivity are

3 $$\begin{aligned} &\rho _{xx}\equiv \vec{E}\cdot {\hat{x}}/J=\rho + A_1 m_x^2+A_2m_z \\&\rho _{xy}\equiv \vec{E}\cdot {\hat{y}}/J=A_1 m_xm_y-B_1m_z -B_2. \end{aligned}$$

Magnetoresistance should be the same when the heterostructure is placed upside-down. This is true if the heterostructure is made of achiral materials. In another word, Eq. (2) should be invariant under transformation of $\vec{n} \rightarrow -\vec{n}$. One should not confuse reciprocity condition with inversion symmetry requirement. For an achiral system with reciprocity condition, $\rho$, $A_1$, and $B_1$ must be even functions of $m_z$ while $A_2$, $A_3$, $B_2$, and $B_3$ must be odd. If one defines $\rho =\sum _{n=0}^{n=\infty }\rho _{n} m_z^{2n}$; $A_1=\Delta \rho _1 + \sum _{n=1}^{n=\infty }a_{1n} m_z^{2n}$; $B_1=-R_1-\sum _{n=1}^{n=\infty }b_{1n}m_z^{2n}$; $A_2=\Delta \rho _2m_z + \sum _{n=1}^{n=\infty }a_{2n}m_z^{2n+1}$; $B_2=-R_2m_z-\sum _{n=1}^{n=\infty }b_{2n}m_z^{2n+1}$; $B_3=\sum _ {n=0}^{n=\infty }b_{3n}m_z^{2n+1}$; and keep only the lowest possible terms in these expansions (if the spin dependent scattering is the main source of magnetoresistance and the power in $m_z$ is related to the number of magnons involved in the scatterings, then one should expect higher-power terms negligible) the longitudinal resistivity and transverse resistivity take following universal forms

4 $$\begin{aligned}&\rho _{xx}=\rho _0 + \Delta {\rho _1}{m_x}^2 + \Delta {\rho _2}{ m_z}^2 \\&\rho _{xy}=Rm_z + \Delta {\rho _1}{ m_x}{m_y}, \end{aligned}$$

where $\rho _0$, $\Delta \rho _1$, $\Delta \rho _2$, and $R \equiv (R_1+R_2)$ are angular-independent material parameters.

To further see that Eq. (4) has exactly the forms of observed UAMR, we consider $\rho _{xx}$ and $\rho _{xy}$ for $\vec{M}$ ($\vec{m}$) varying in the *xy*- and *yz*-, and *zx*-planes and define angle $\alpha$ between the magnetization $\vec{M}$ and *x*-axis when $\vec{M}$ rotates in the *xy*-plane; or angle $\beta$ ($\gamma$) between $\vec{M}$ and the *z*-axis when $\vec{M}$ rotates in the *yz*-plane (*zx*-plane). We then have the usual anisotropic magnetoresistance $\rho _{xx}=\rho _0+ \Delta \rho _1 \cos ^2\alpha$ and planar Hall resistance $\rho _{xy}=( \Delta \rho _1/2)\sin (2\alpha )$ for $\vec{M}$ in the *xy*-plane; newly discovered UAMR $\rho _{xx}=\rho _0+\Delta \rho _2\cos ^2\beta$ and accompanied unusual angular dependence of planar Hall resistivity $\rho _{xy}=R\cos \beta$ for $\vec{M}$ in the *yz*-plane; and $\rho _{xx}=\rho _0+\Delta \rho _2+(\Delta \rho _1-\Delta \rho _2)\sin ^2 \gamma$ and $\rho _{xy}=R\cos \gamma$ for $\vec{M}$ in the *zx*-plane. Clearly, both phases and amplitudes of $\rho _{xx}$ and $\rho _{xx}$ are what were observed in experiments^3–8,10–14^. Thus, UAMR does not need to involve the spin-Hall and the inverse spin-Hall effects. In some bilayers such as $\textrm{Pt}| \textrm{Y}_{3}\textrm{Fe}_{5}\textrm{O}_{12}$^9^, an additional three-fold angular dependence of $\rho _{xy}$ on $\beta$ were observed. This is also allowed in Eq. (3) if we keep the second term in the expansion of $B_2$ and redefine $\Delta \rho _3\equiv b_{22}$ such that $\rho _{xy}=R\cos \beta +\Delta \rho _3\cos ^3\beta$.

Real bilayer heterostructures show various possible relative values of $\Delta \rho _1$ and $\Delta \rho _2$. In $\textrm{Pt}|\textrm{Fe}_{3}\textrm{O}_{4}$ where a polycrystalline $\textrm{Pt}$ is on a single crystal poor metal $\textrm{Fe}_{3}\textrm{O}_{4}$, $\Delta \rho _2\simeq 0$ was observed^12^ such that $\rho _{xx}$ is not sensitive to $\beta$, or rotating magnetization in the *yz*-plane (perpendicular to the current). This observation is difficult to understand within the SMR theory, but is a natural outcome of the case of $\Delta \rho _2\simeq 0$ in the present theory. The slightly different angular dependence of usual AMR curve can be naturally understood from the contribution of the poor single crystal metal of $\textrm{Fe}_{3}\textrm{O}_{4}$^15^. Whether $\Delta \rho _2$ is an intrinsic parameter of materials is an interesting question. It should not be difficult to settle this issue. One needs only to fabricate similar bilayers under different conditions so that the interface and microscopic structures of two layer would be different. All samples should have the same $\Delta \rho _2$ if it is intrinsic, and different $\Delta \rho _2$ otherwise. In $\textrm{W}|\textrm{CoFeB}|\textrm{MgO}$ structure, $\rho _{xx}$ is not sensitive to $\gamma$, or rotating magnetization in the *xz*-plane. This is the consequence of $\Delta \rho _1\simeq \Delta \rho _2$. To our knowledge, the case of $\Delta \rho _1\simeq 0$ is yet to be observed. The current theory explains also why three amplitudes of the oscillations of $\rho _{xx}$ with $\alpha$, $\beta$, and $\gamma$ satisfy following rule: sum of the two’s equal to the third one.

As shown above, UAMR can be perfectly understood from the combined actions of magnetism and interfacial field of a bilayer on electron transport. According to this mechanism, the UAMR should decrease with thickness of polycrystalline metal film when the film thickness is larger than interacting range of the interfacial field such that shunting effect dominates and resistance from interface region is less important. One should also expect that UAMR decreases when the film thickness is smaller than the interface-roughness in structure and physical properties such that interfacial field characterized by $\vec{n}$ becomes problematic and resistance cannot feel $\vec{n}$ effect. This may explain why UAMR is peaked at an optimal film thickness. One likely interface effect on the resistance is from the interfacial scatterings. If the scatterings involve the interfacial phonon, then UAMR should be more pronounced at high temperature because a phonon scattering is more effective. All these features are shared by UAMR experiments^3–11,14^. Thus the thickness-dependences of UAMR provides the information of interacting range of the interfacial potential or interfacial field. The optimal thickness, at which the UAMR is most pronounced, contains information of interface roughness. So far, UAMR are found in bilayers of non-magnetic film on a magnetic layer. From the theory presented in this paper, similar results should exist in bilayers of two different magnetic layers that could be both magnetic polycrystalline metals or one polycrystalline metal and one magnetic insulator. If one uses strong magnetic field to align the magnetisations of the two magnetic layers in the same direction in angular dependence measurement of MR, then the above analysis is applicable. Otherwise, one should generalize the theory into the case of three vectors. In a short summary, there are experimental ways to distinguish the current theory from the popular SMR theory. One important remark is to exclude the metallic single crystal that will introduce extra contributions to the angular dependences of magnetoresistance^15^.

In SMR theory, there are five material parameters to be extracted from various experimental measurements. They are conductivity at bilayer interface, spin Hall angle and spin diffusion length of the heavy metal, and complex spin-mixing conductance (real and imaginer parts). These five parameters cannot be directly extracted from transport measurements, and are obtainable by fitting thickness dependences of conductivity to certain theoretical models plus other independent experiments such as ferromagnetic resonance on the same bilayers^5^. In comparison, there are only four material parameter in this theory. These four parameters defined in Eq. (4) as $\rho _0$, $\Delta \rho _1$, $\Delta \rho _2$, and *R* can directly be extracted from resistivity measurements. The current theory predicts also an angular dependence of $\rho _{xz}\equiv \vec{E}\cdot {\hat{z}}/J=-Rm_y + \Delta \rho _4 m_xm_z$. This is highly non-trivial: It says that a voltage drop exist in the *z*-direction proportional to $m_y$ and $m_xm_z$. This prediction distinguishes the current theory with all other existing theories. Of course, how to find a proper system that allows one to measure a small voltage drop perpendicular to an ultra-thin film may not be easy.

It should be emphasised that a complete theory is presented here with precise predictions of how MR should vary in three mutually perpendicular planes, and how their amplitudes should be related to each other. Several very feasible experiments are proposed to test this theory against the SMR theory. The theory is so general that any achiral system, no matter how complicate its microscopic Hamiltonian and interactions might be, must agree with the results presented here. The approach is similar to the derivation of Einstein’s gravitation theory that is about possible construction of a rank 2 tensor out of metric tensor^20^. Of course, UAMR is not as fundamental as gravitation theory, and our results are a relationship between MR and magnetization, not a partial differential dynamical equation as the Einstein’s gravitation theory is.

It should also be interesting to study chiral heterostructure when reciprocity condition does not hold. Follow exactly the analysis above, it is easy to see that UAMR of chiral system should be described as $\rho _{xx}=\rho _0 +R_1m_z \Delta \rho _1m_x^2 + \Delta \rho _2 m_z^2$ and $\rho _{xy}=Rm_z + \Delta \rho _1 m_xm_y+ \Delta \rho _4 m_z^2$. $\beta$ and $\gamma$ dependences of both $\rho _{xx}$ and $\rho _{xy}$ are different from its achiral counterparts while the $\alpha$ dependence are the same. It should be very interesting to test these predictions using heterostructure made of helimagnets.

Onsager reciprocal relation breaks down for our bilayers involving magnetization since the magnetic materials do not respect reversibility. Of course, extra symmetries can impose further restriction on the possible structure of the resistivity tensor. It should also be pointed out that this manuscript and Ref. 15 consider different systems although they share the similar mathematical technique and ask similar question. Reference 15 study the angular dependence of resistance of homogeneous samples such as ferrimagnets or antiferromagnets described by at least two order parameters. On the other hand, this manuscript consider bilayers heterostructure with at least one magnetic layer. The objective is to provide a natural explanation for the universal angular dependences in three mutually orthogonal planes. Both layers could be metallic, or one metallic and one insulating. Both of them can be magnetic, or one magnetic and one non-magnetic.

In conclusion, unusual angular dependence of magnetoresistance in bilayers consisting of one non-magnetic film and one magnetic film can be explained by a two-vector resistance theory if the metallic layer(s) is polycrystalline. It is predicted that similar behaviours should also hold for bilayers of two different magnetic films. The general UAMR can come from physics beyond the spin-Hall and inverse spin-Hall effects. Experiments that can test this theory are also proposed.

## Data Availability

All data generated or analysed during this study are included in this published article.
